# Test-Retest Reliability of a Physical Activity Behavior, Health and Wellbeing Questionnaire in Adolescents

**DOI:** 10.12688/openreseurope.16535.2

**Published:** 2024-06-28

**Authors:** Padraic Rocliffe, Ian Sherwin, Patricia Mannix-McNamara, Ciaran MacDonncha, Brendan T. O' Keeffe

**Affiliations:** 1 Physical Education and Sport Sciences, University of Limerick, Limerick, County Limerick, V94 T9PX, Ireland; 2 School of Education, University of Limerick, Limerick, County Limerick, V94 T9PX, Ireland; 3 Faculty of Education, Western Norway University of Applied Sciences, Bergen, Norway, 28, 5063, Norway

**Keywords:** School; Physical Activity Behavior; Health; Wellbeing; Youth; Reliability.

## Abstract

**Background:**

The aim of this study was to examine the test-retest reliability of the physical activity behavior, health and wellbeing questionnaire, in adolescent populations, administered by teachers in school settings, in the Republic of Ireland.

**Methods:**

A cross-sectional, mixed sample of 55 participants (45.5% males: Age, 13.94 (±.40) years) were included. The participants completed the questionnaire on two occasions (T1 and T2), on the same day and time, one week apart following identical procedures. Variables for testing included physical activity behavior (n=13), health (n=11) and wellbeing (n=2). Test-retest reliability of the questionnaire’s covariates, including family affluence and physical impairments were also examined.

**Results:**

Systematic error (Bland-Altman plots) was found to be near to zero for each of the physical activity behavior, health and wellbeing variables. The combined mean coefficient of variation was lower for females (10.19%) in comparison to males (13.01%). The combined mean intraclass correlation coefficients were higher for females (0.901) than males (0.822). Similarly, the combined mean Cronbach alpha coefficient were higher for girls (0.908) than boys (0.821).

**Conclusions:**

This study found the physical activity behavior, health and wellbeing questionnaire to be reliable for use in adolescent populations.

## Plain language summary

The goal of this study was to assess whether a questionnaire about physical activity behavior, health, and wellbeing is reliable when administered by teachers to adolescents in schools. A group of 55 participants with mixed genders (45.5% males) and an average age of 13.94 years took part. They were asked to complete the questionnaire twice, one week apart, following the same rules.

The questionnaire covered various aspects of physical activity behaviour, health, and wellbeing e.g., moderate to vigorous physical activity, sedentary behavior, body mass index and life satisfaction as well as extra factors known to impact adolescent health such as family background and physical impairments. The results showed that the responses to the questions remained quite stable over time. Statistical analyses were conducted, and they indicated minimal changes in the answers when the same questions were asked again after one week.

Interestingly, the reliability was slightly better for females compared to males. This difference might be attributed to variations in communication styles or willingness to disclose among different genders. Overall, the questionnaire is consistent and can be utilized to gain insights into how adolescents perceive their physical activity, health, and wellbeing.

## Introduction

Health authorities, researchers, and government officials use national monitoring of health behaviors as a crucial tool for policymakers to track outcomes related to health promotion ( Ng *et al.*, 2019). Physical activity is a powerful predictor of future health among adolescent populations, impacting indicators of physical fitness (e.g., aerobic capacity, flexibility, strength), obesity (e.g., weight, body mass index, skinfolds), negative mental health outcomes (e.g., depression and anxiety) and positive mental health outcomes (e.g., life satisfaction and wellbeing) ( Laurson *et al.*, 2015; McMahon *et al.*, 2017; Monshouwer *et al.*, 2013; Ruiz *et al.*, 2009). Therefore, the monitoring of physical activity behaviors for future health may be of significant importance. Physical activity is described as “any bodily movement produced by skeletal muscle that results in energy expenditure” ( Caspersen *et al.*, 1985 p126). Globally, physical activity recommendations advocate for an average of 60 minutes, at a moderate to vigorous intensity, across a week, for adolescent populations ( World Health Organization, 2022a). Despite this, worldwide trends indicate that approximately four in every five adolescents are failing to meet these recommendations ( World Health Organization, 2022b). In addition, physical inactivity is understood to track from adolescents into adulthood and thus is having a profound effect on global health costs which are forecast to reach $300 billion by 2030 ( World Health Organization, 2022c). Furthermore, higher levels of physical activity have been found to be associated with higher levels of physical fitness and wellbeing and reduced risk of disease such as obesity and depression ( Abdukic, 2015; Barney *et al.*, 2019; Costigan *et al.*, 2015; Farias *et al.*, 2015; Muntaner-Mas & Palou, 2017). Therefore, monitoring indicators of physical activity, health and wellbeing may be of significant importance to both enhancing adolescent health and reducing global costs of physical inactivity.

Schools are recognised as primary institutions to advocate for adolescent health ( Cooper *et al.*, 2010; Morton *et al.*, 2016) by “constantly strengthening its capacity as a healthy setting for living, learning and working” ( ISPAH, 2020; World Health Organization, 2022c p1). Physical education programs foster physically active environments thought to enhance factors of adolescent health ( Pate *et al.*, 2006; Rocliffe *et al.*, 2023a; Rocliffe *et al.*, 2023b). Despite this, the prevalence of obesity, which is considered to be “abnormal or excessive fat accumulation which may impair health”, has been found to impact over 340 million adolescents worldwide ( Chiyanika *et al.*, 2020; Sinha *et al.*, 2020 p738). Furthermore, the relationship between obesity as a predictor of various other health indicators e.g., type 2 diabetes, is well established ( Buffart *et al.*, 2008; Institute of Medicine, 2012). Similar to physical activity, levels of obesity are known to track into adulthood, which provides further impetus to measure body mass during this phase of life ( Silva *et al.*, 2013). Health related physical fitness comprises an array of components such as cardiorespiratory fitness, musculoskeletal fitness and body composition that are understood to be strong indicators for future health ( Hurtig-Wennlof *et al.*, 2007; Ortega *et al.*, 2008; Smith *et al.*, 2014). Hogstrom *et al.,* found a strong correlation between low cardiorespiratory fitness and early mortality in a sample of 700,000 adolescents ( 2016), while muscular strength is consistently cited as protective mechanism to combat obesity and cardiometabolic risk factors such as blood pressure ( Garcia-Hermoso *et al.*, 2019). In addition, although there is a paucity of evidence regarding blood pressure in adolescent populations, correlations indicate a strong relationship between blood pressure that is high and adolescent sedentary behavior ( Martinez-Gomez *et al.*, 2009). The predictive capacity of obesity on health indicators and health related physical fitness, as a measure of future health, further illuminates the requirement to monitor these variables during adolescence ( Kaminsky *et al.*, 2013).

Prevalence of negative mental health outcomes e.g., depression and anxiety account for 45% of the burden of disease globally, in adolescent cohorts ( Gore *et al.*, 2011). According to the World Health Organization, depression is expected to impact 322 million people worldwide by 2030, illuminating it as a key risk factor for disability ( 2017). In parallel, anxiety is considered the sixth leading risk factor for disability, impacting an estimated 265 million individuals worldwide ( Kessler *et al.*, 2007). The onset of depression and anxiety are most prevalent in adolescents ( Kessler *et al.*, 2007). Thus, accurately monitoring such health behaviors during these years, to adequately inform policy development and to combat mental ill health is pertinent. Wellbeing and life satisfaction are considered integral indicators of positive mental health ( Dienlin & Johannes, 2020). Both have been found to decline during adolescence ( Costigan *et al.*, 2015; Mendez-Gimenez *et al.*, 2022; Proctor *et al.*, 2009) and are inherently linked with depression and anxiety that tracks in to adulthood with research suggesting a bi-directional relationship i.e., a decrease in negative mental health outcomes (e.g., depression and anxiety) when wellbeing and life satisfaction are high and an increase when they are low ( Guney *et al.*, 2010; Seo *et al.*, 2018). In addition, worldwide health costs associated with mental ill health such as depression have reached $9.9 billion annually ( Cruz, 2012). This evidence suggests the immediate requirement to track indicators that are strongly associated with public health promotion, to alleviate this economic burden.

Objective measures of physical activity, health and wellbeing in large cohorts of school going adolescents, paralleled with the associated costs which often limit sample sizes, require significant time and the need for an adequately qualified testing team that are often not feasible ( Lam *et al.*, 2022; Muntaner-Mas *et al.*, 2019). In the context of the current study, self-report measures provide a suitable alternative as costs are significantly lower, they do not take a great deal of time to administer and are more easily accessible to a wider cohort of schools ( Sprengeler *et al.*, 2019). However, it is imperative that such self-report measures are rigorously assessed to enhance the validation of clinimetric tools as demonstrated by Navarro-Flores *et al*. (2022) and Ahmadnezhad *et al*. (2023). Validity and reliability are key components of this process. Validity is defined as “the extent to which a concept is accurately measured in a quantitative study” ( Heale & Twycross, 2015 p66). Extensive research has been conducted on the validity of a high proportion of the variables used in the current study ( Barnett *et al.*, 2015; Bervoets *et al.*, 2014; Hardie Murphy *et al.*, 2015; McKay & Andretta, 2017; Ramirez-Velez *et al.*, 2017; Ringdal *et al.*, 2018; Saunders *et al.*, 1997; Shannon *et al.*, 2020). Reliability refers to “the reproducibility of assessment data or scores, over time or occasion ( Downing, 2004 p1006). While there is some research that confirms the reliability of the variables utilized in the current study ( Haugland & Wold, 2001; Levin & Currie, 2014; Nagai *et al.*, 2018), there is a paucity of evidence examining the test-retest reliability of tools to estimate all components of physical activity behavior, health and wellbeing as part of one questionnaire, administered by teachers in school settings. Therefore, the aim of the current study was to examine the test-retest reliability of the physical activity behavior, health and wellbeing (PABHAW) questionnaire in adolescent populations. It is hypothesized that the physical activity behavior, health and wellbeing questionnaire will demonstrate high test-retest reliability when administered to adolescent cohorts.

## Methods

### Ethics approval

Research ethics approval (2022_09_15_EHS) for this study and the associated protocols was granted by the research ethics committee of the Faculty of Education and Health Sciences, University of Limerick, Ireland. Informed consent was received as follows:

1. Opt out consent forms were sent to the parents of the participants via the school communication channels in which they needed to sign should they not wish their adolescent to participate.
2. Informed consent was embedded in the PABHAW web link and was obtained by checking the appropriate box. All participating adolescents needed to check this box in order to participate.

### Participants

Research ethics approval for this study and the associated protocols was granted by the research ethics committee of the Faculty of Education and Health Sciences, University of Limerick, Ireland. Data collection was conducted between September and November 2022. A convenience sample of 55 adolescents (25 males, 30 females), were recruited from three mixed-sex secondary schools located in the southern region of the Republic of Ireland. All second-year class groups (13–14 years) from each school were invited to participate. The PABHAW questionnaire took approximately 35 minutes to complete. Second-year class groups were deemed the most appropriate to provide accurate identification of PABHAW patterns in adolescents due to both their developmental stage and availability to participate due to less engagement in examinations that were pertinent to other year groups during the data collection period. The initial sample consisted of 85 participants (41 males, 44 females). However, in accordance with item nine of the physical activity questionnaire for adolescents (PAQ-A), students who were sick within the last seven days before completing the questionnaire were removed. It was considered that inclusion of these participants may introduce bias to the dataset e.g., a sick participant at T1 but a non-sick participant at T2 ( Kowalski *et al.*, 2004). Thus, in order to limit confounding factors such as this, these participants were excluded. All participants were provided with unique identifier codes to ensure anonymity. Demographics details obtained included gender, age, year group, nationality and jurisdiction i.e., rural or urban ( Table 1).

**Table 1.** Demographic characteristics of participants.

(N=55)	
**Gender**	Males (45.5%) Females (54.5%)
**Age**	13.94 (±.40) Years
**Nationality**	Republic of Ireland (87.3%) Outside of Ireland (12.7%) (America, United Kingdom, Lithuania, Ukraine).
**Location**	Urban (40%) Rural (60%)

### Procedure

The Irish education system encompasses three tiers: primary school (5–12 years), secondary school (12–18 years) and third-level institutes (18+). This cross-sectional study utilized Qualtrics online software to distribute the PABHAW questionnaire to participants in secondary school. First, an invitation to participate outlining the aims and objectives of the study was circulated to the school principals, parents and participants to obtain consent. Second, the PABHAW questionnaire links for Test 1 (T1) and Test 2 (T2) were distributed to the head physical education teacher for completion by the participants during timetabled physical education class. Informed consent was embedded in to the PABHAW questionnaire web link and was indicated by checking the appropriate box. Participants were permitted to exit the PABHAW questionnaire web link at any point should they have wished to depart the study. Two individual data collection points (T1 and T2) were conducted on the same day and time, one week apart following identical procedures. Non-response bias was minimized where possible, utilising both a reminder email and a detailed standard operating procedure to the head physical education teachers 10 days in advance of administering the questionnaire for T1. Head physical education teachers conducted one familiarization trial of the PABHAW questionnaire and were given opportunities to engage with the lead researcher on the project regarding any clarifications they may have had. Participants were required to answer each question in the PABHAW questionnaire to minimise unanswered questions and subsequent missing values. The skip logic function was utilised for the items on perceived physical competence ( Harter, 1982) which accounted for some missing values. The total allocated time to complete the questionnaire was set at 53-minutes or one single period of physical education.

### Development of the PABHAW questionnaire

The PABHAW questionnaire was assembled using variables with established validity and reliability. The questionnaire comprises multiple choice, ordinal scale, interval scale, ratio scale, open and closed ended question types. Participants response formats aligned with each question type. Content validity was established via an expert stakeholder group of 12 participants in the first instance and a further 60 second year adolescents (62% males) in the second instance, who piloted the PABHAW questionnaire. Inclusion of the expert stakeholder group was underpinned by their level of expertise in the area of physical activity behavior, health and wellbeing and included head physical education teachers (n=3), experts in the field (n=4) and relevant postgraduate students (n=5) assigned to a specific field of study included in the questionnaire (e.g., physical activity). The inclusion of the pilot sample of adolescents emulated the target participant profile for the study. A 5-point Likert scale to evaluate the PABHAW questionnaire (1=unclear, 5=clear) was utilized. The expert panel and adolescent groups were provided with opportunities to record the duration to complete each section of the PABHAW questionnaire in parallel with reporting the relevancy and flow of the questionnaire items. Aligning with thresholds underpinned by O’Keeffe *et al.* (2019), any clarity ratings below 3 were amended or were completely removed. PABHAW questionnaire items with an evaluation score of below 3 were amended. The final draft of the PABHAW questionnaire was approved by the two lead authors (PR, CMD) and edits were resolved via consensus. Extensive research has been conducted on the validity of many of the variables used in the current study ( Barnett *et al.*, 2015; Bervoets *et al.*, 2014; Hardie Murphy *et al.*, 2015; McKay & Andretta, 2017; Ramirez-Velez *et al.*, 2017; Ringdal *et al.*, 2018; Saunders *et al.*, 1997; Shannon *et al.*, 2020). Standardised protocols are detailed in the following paragraphs. The PABHAW questionnaire can be sourced via the extended data included in this manuscript.

### Physical activity behavior

Physical activity behavior was estimated using 13 variables most of which had pre-established validity and reliability ( Burns, 2012; Godin & Shepard, 1986; Hardie Murphy *et al.*, 2015; Harter, 1982; Kowalski *et al.*, 2004; Motl *et al.*, 2001; Nagai *et al.*, 2018; Prochaska *et al.*, 2001; Prochaska *et al.*, 2002; Reynolds *et al.*, 1990; Sallis *et al.*, 1997; Saunders *et al.*, 1997; Wu *et al.*, 2011). Variables were chosen to give a comprehensive measure across a range of validated tools pertaining to both levels of physical activity (physical activity, moderate to vigorous physical activity, sedentary behavior) and indicators of physical activity behavior (intention to be physically active, enjoyment of school/physical education, social support, self-efficacy, perceived physical competence). Physical activity over the last seven days was estimated via a modified version of the physical activity questionnaire for adolescents (PAQ-A) ( Kowalski *et al.*, 2004). Similar to Aggio *et al.*, item one of the PAQ-A was modified to reflect “activities deemed more representative to the study population” ( 2016). Therefore, activities were gleaned from a recent national audit on typical school provision of physical education, physical activity and sports in the Republic of Ireland ( Rocliffe *et al.*, 2023c). Similar modifications are recommended when utilizing the PABHAW questionnaire in the international context. Participants completed eight items relating to physical activity during their spare time, physical education, lunch, right after school, evening, weekends and physical activity across each day over the last week using 5-point Likert scales. All eight items were then summed and divided by eight to formulate a final PAQ-A score between one and five. A score of one represented low physical activity while a score of five indicated high physical activity. Item nine referred to sickness during the last week that prevented one to partake in physical activity. Participants that indicated “yes” to this item were excluded from the analysis. In addition, this item was utilized as one of three covariates in the questionnaire.

Moderate to vigorous physical activity (MVPA) was estimated via the two item PACE+ questions ( Prochaska *et al.*, 2001). Participants reported the number of days (0-7) they were physically active for at least 60 minutes per day in the past seven days for item one and the number of days (0-7) they were physically active for at least 60 minutes per day over a typical or usual week. Items were summed and divided by two to get the mean MVPA score. Sedentary behavior was estimated via a modified version of the Self-Administered Physical Activity Checklist (SAPAC) ( Sallis *et al.*, 1996) and a range of the sedentary behavior items utilized in the Determinants of Diet and Physical Activity Knowledge Hub (DEDIPAC) ( Brug *et al.*, 2017). Participants completed 12 items relating to minutes spent sedentary on an average weekday and an average weekend, using a 7-point Likert scale, ranging from “0 minutes per day” to “about or more than 4 hours per day”. Items were summed and divided by 12 to get a mean score for sedentary behavior on weekdays and weekends. An overall sedentary behavior score was derived by adding the mean weekday and weekend scores and dividing by two.

The intention to be physically active scale ( Godin & Shepard, 1986), consisted of a one item 5-point Likert scale ranging from “I am sure I will not be physically active” to “I am sure I will be physically active”. Enjoyment of physical education was estimated using a one item 5-point Likert scale ranging from “disagree a lot” to “agree a lot” ( Motl *et al.*, 2001). Enjoyment of school was estimated using a simple tool developed by the researcher. Using the same methods pertaining to the enjoyment of physical education scale, participants indicated their enjoyment of school on a 5-point Likert scale ranging from “disagree a lot” to “agree a lot”. Social support to engage in physical activity was estimated using the social influences scales ( Reynolds *et al.*, 1990). Participants completed items related to 1) physical activity with friends, 2) peer support and 3) family support, via a mix of 5-point Likert scales and “yes”, “no” questions. Each individual variable was summed to provide a single score. For the variable concerning peer support, item 1; “do you encourage your friends to do physical activities or play sports” was excluded from the analysis in line with standardised guidelines.

Self-efficacy, which initially was a 17-item scale ( Reynolds *et al.*, 1990; Sallis *et al.*, 1992), was modified to measure support seeking, positive alternative and barriers ( Saunders *et al.*, 1997). Motl *et al.* (2000) further refined the scale resulting in an eight item, 5-point Likert scale ranging from “disagree a lot” to agree a lot”. Items were then summed with higher scores meaning higher self-efficacy and lower scores indicating lower self-efficacy. Similar to Burns (2012), perceived physical competence was estimated using a subscale from the perceived competence scale for children ( Harter, 1982). The participants were presented with items in which they chose which adolescent they were most like e.g., “I do very well at all kinds of games and sports” versus “I don’t feel that I am very good when it comes to games and sports”. The participant then indicated if this statement is “really true for me” or “sort of true for me”. Items were then summed with higher scores indicating higher levels of perceived physical competence and lower scores indicating lower perceived physical competence. The second covariate referred to family affluence and was estimated via the family affluence scale ( Currie *et al.*, 1997). Participants completed 6 items with a mix of 3 and 4-point Likert scales and “yes”, “no” questions. Items were summed to provide a composite score. Higher scores indicated higher family affluence; lower scores indicated lower family affluence. The third covariate referred to functional difficulties regarding mobility and was estimated using the UNICEF/Washington Group Child Functioning Module ( Crialesi *et al.*, 2016). Just one item from the UNICEF/Washington Group Child Functioning Module, was deemed necessary to establish functional difficulties regarding mobility, therefore, validity was not assumed. Participants reported their level of difficulty walking 500 metres via a 4-point Likert scale ranging from “cannot do at all” to “no difficulties”.

### Health

Health indicators included physical fitness, subjective health complaints and body mass index (BMI). Physical fitness was estimated via the International Fitness Scale and has established validity and reliability ( Ortega *et al.*, 2011; Ramirez-Velez *et al.*, 2017). The self-report questionnaire includes five components of health-related fitness: general fitness, cardiorespiratory fitness, muscular strength, speed/agility and flexibility. Participants completed each item using a 5-point Likert scale ranging from “very poor” to “very good”. Subjective health complaints were estimated via the Health Behavior in School-Aged Children symptom checklist and is a non-clinical measure of subjective health in the last six months and has established validity and reliability ( Gariepy *et al.*, 2016; Haugland & Wold, 2001; Heinz *et al.*, 2022; Heinz *et al.*, 2020; Potrebny *et al.*, 2019). There are a total of eight variables including headache, stomach ache, backache, feeling low, irritability or bad temper, feeling nervous, difficulties in getting to sleep and feeling dizzy. Participants completed each item using a 5-point Likert scale ranging from “rarely or never” to “about everyday”. In accordance with Potrebny *et al.* (2019) a somatic health complaints (headache, stomach ache, backache, feeling dizzy) and psychological health complaints (feeling low, irritability/bad temper, feeling nervous, difficulties sleeping) score was obtained by summing each and dividing by four. In addition, somatic and psychological health complaints were summed to formulate an overall continuous variable resulting in a scale of 0 (no symptoms at all) to 32 (maximum symptom load). Participants were furnished with a BMI protocol that instructed a parent/guardian to conduct anthropometric measures of the participants height to the nearest 0.1cm and weight to the nearest 0.1kg at home using measuring tape and weighing scales, three days prior to completing the PABHAW questionnaire. The participants subsequently recorded their height and weight in the PABHAW questionnaire on the day of testing. During the measurements participants were instructed to remove their shoes and wear light clothing. Participant weight (kg) was divided by height (m ^2^) to formulate a BMI score. The International Fitness and subjective health scales are found to have good validity and reliability in adolescent populations. 

### Wellbeing

Wellbeing was estimated via the Warwick-Edinburgh mental wellbeing scale ( Tennant *et al.*, 2007). Life Satisfaction was estimated using Cantril’s Self-Anchoring Striving Scale ( Cantril, 1965). Both measures have good validity and reliability in adolescent populations ( Levin & Currie, 2014; Mazur *et al.*, 2018; McKay & Andretta, 2017; Ringdal *et al.*, 2018; Shannon *et al.*, 2020). For the Warwick-Edinburgh mental wellbeing scale, participants were asked to respond to a 14-tem positively worded scale, with five response categories that ranged from “none of the time” to “all of the time” and were summed to provide a single score. Summed scores ranged from 14–70 with higher scores indicated higher wellbeing; lower scores indicated lower wellbeing. Cantril’s Self Anchoring Striving Scale consists of a ladder with steps numbered from 0–10 whereby 10 represents the best possible life for the participant and 0 represents the worst possible life for the participant. A score of eight and above indicated that the participant is thriving i.e., wellbeing is strong, consistent, and progressing. A score of 5–7 indicated that the participant is struggling i.e., wellbeing that is moderate or inconsistent, while a score of four and below indicates the participant is suffering i.e., their wellbeing is at high risk.

### Analysis

IBM Statistical Package for the Social Sciences 28 (SPSS) was utilized to analyse the data collected from the study with *p*<0.05 as a criterion for statistical significance. The data was downloaded from Qualtrics and imported to SPSS to conduct data analysis upon completion of data collection. For the purpose of this study, incomplete data was defined as having 10% or more of the PABHAW questionnaire incomplete. Subsequently, all 55 data points were deemed complete for analysis. Descriptive statistics for time point 1 (T1) and time point 2 (T2), including means, standard deviations and intertest differences were calculated for each variable. Higher mean values most often indicated higher (better) scores in a variable, however, in some cases higher mean values indicated lower (worse) scores e.g., sedentary behavior, BMI and health complaints. Reliability was examined via relative and absolute indices. Relative reliability refers to the extent to which individuals consistently maintain their position within a sample when subjected to repeated measures, while absolute reliability “is the degree to which repeated measurements vary for individuals” ( Bruton *et al.*, 2000 p95). Relative reliability was assessed and illustrated via intraclass correlation coefficients while absolute indices were examined via intertest differences, coefficient of variation and limits of agreement. Paired-samples *t*-tests were used to determine if there were statistically significant systematic and random mean differences between T1 and T2. Variables were examined for the existence of outliers. A sensitivity analysis was undertaken to examine the effects of any outliers in the data by conducting a paired-samples *t*-test with and without outliers. Some variation in *p* values were found; however, overall significance trends did not change. To respect the required assumption for the paired-samples *t*-test and to also moderate the impact on other assumptions (e.g., normality), outliers greater or lesser than 3 standard deviation points were removed. A Shapiro Wilk test and visual inspection of the associated histograms, Q-Q plots and box plots were used to test for normality ( Razali & Wah, 2011; Shapiro & Wilk, 1965). Where assumptions for normality were not met, a nonparametric Wilcoxon signed-rank test was utilized to confirm the findings of the paired-samples *t*-tests. While the paired-samples *t*-tests are based on the assumption of normal distribution, Mehta *et al.* (2018) indicates that “this assumption is not necessary for the valid estimation of intraclass correlation” in analysis that does not make population inferences such as in the case with test-retest reliability data. Therefore, an intraclass correlation coefficient was used to assess the consistency of agreement between T1 and T2 using a 2-way mixed effects, absolute agreement, average measure model. Aligning with thresholds underpinned by Koo and Li (2016) <.50 was classified as poor agreement, 0.50–0.75 moderate agreement, 0.75–0.90 good agreement and >0.90 excellent agreement. Internal consistency was assessed using Cronbach alpha coefficients.

Bland-Altman plots were used to graphically evaluate and illustrate agreement between T1 and T2. In order to express the Bland-Altman plots quantitatively, the following formula was utilized to establish the coefficient of variation (CV) between T1 and T2: (Standard Deviation / Mean) *100. In addition, O’Keeffe *et al.* (2020 p51) notes “the advantage of using a dimensionless statistic such as the CV to facilitate comparison of reliability between different measurement tools or different groups” such as in the current study. Considering the broad range of variables included in the PABHAW questionnaire and that the “threshold for acceptable percentage error should be specific to the variable being measured” ( O’Keeffe *et al.*, 2020 p51), it was deemed inappropriate to specify thresholds with which the CV data was considered reliable. The 95% limits of agreement for each PABHAW variable were established by calculating the intertest mean difference ±1.96 of the intertest differences ( Atkinson & Nevill, 1998). Using the Bland-Altman plots, a visual inspection of the measurement differences (T2-T1) against the means was conducted to identify if heteroscedasticity was present. All data were analysed for males and females separately.

## Results

For the purpose of analysis, the sample were split into males (n=25) and females (n=30). Descriptive statistics including means and standard deviations for T1 and T2, and intertest differences (T2-T1) are provided in Table 2. Intertest differences were zero or close to zero for all variables in both males and females with the exception of life satisfaction in males (Mean difference (SD) = -1.79 ( *±* 6.74)), self-efficacy in males (MD = -1.64 ( *±* 5.57)) and females (MD = -1.10 ( *±* 4.04)) and overall health complaints in females (MD = -1.79 ( *±* 3.65)). A paired-samples *t*-test ( Table 3) indicated no statistically significant intertest differences in either group ( *p* <0.05), aside from, BMI (MD = -0.62 ( *±* 1.12), *p* = 0.007), psychological health complaints (MD = -0.25 ( *±* 0.52), *p* = 0.017), cardiorespiratory fitness (MD = 0.32 (± 0.66 *, p* = 0.017 *)* and overall health complaints in females (MD = -1.79 ( *±* 3.65), *p* = 0.013). It must be note that inferences of reliability are not taken from the paired sample t-test findings (both significant and non-significant) due to small sample sizes. The intraclass correlation coefficient values ranged from 0.504 (cardiorespiratory fitness) to 0.993 (BMI) in the male group and from 0.676 (flexibility) to 1.000 (physical impairment) in the female group ( Table 3). The mean intraclass correlation coefficients were higher for females (0.901) than males (0.822).

**Table 2.** Test-retest measurements for the physical activity behavior, health and wellbeing in male and female groups.

	Males (n=25)				Females (n=30)			
	Test 1 Mean (SD)	Test 2 Mean (SD)	Shapiro-Wilk	Mean Differences T2 – T1 (SD)	Test 1 Mean (SD)	Test 2 Mean (SD)	Shapiro-Wilk	Mean Differences T2 – T1 (SD)
**Physical Activity ** **Behavior Variables**								
PAQ-A	3.07 (0.68)	3.07 (0.70)	0.074	0.00 (0.51)	2.75 (0.77)	2.77 (0.76)	0.600	0.02 (0.35)
MVPA	4.88 (1.76)	4.94 (1.83)	0.080	0.06 (1.39)	3.87 (1.75)	4.07 (1.64)	0.030	0.20 (0.77)
Sedentary Behavior Weekday	2.83 (0.50)	2.85 (0.59)	0.594	0.02 (0.47)	2.76 (0.51)	2.76 (0.44)	0.331	0.01 (0.29)
Sedentary Behavior Weekend	2.87 (0.51)	2.93 (0.69)	0.037	0.06 (0.52)	2.90 (0.61)	2.85 (0.57)	0.143	-0.05 (0.41)
Overall Sedentary Behavior	2.86 (0.49)	2.91 (0.63)	0.252	0.05 (0.47)	2.85 (0.53)	2.81 (0.49)	0.380	-0.04 (0.25)
Intention to be Physically Active	4.08 (0.86)	3.68 (1.11)	0.029	-0.40 (1.08)	3.37 (1.10)	3.50 (0.90)	<0.001	0.13 (.73)
Enjoyment of School	3.40 (1.29)	3.68 (1.28)	<0.001	0.28 (1.02)	3.07 (1.22)	3.07 (1.16)	<0.001	0.00 (.85)
Enjoyment of Physical Education	4.48 (0.92)	4.40 (0.87)	0.003	-0.08 (0.64)	4.23 (0.86)	4.27 (1.01)	<0.001	0.03 (0.57)
Social Support (Friends)	5.04 (0.75)	5.04 (0.75)	<0.001	0.00 (0.59)	4.90 (0.80)	4.80 (0.85)	<0.001	-0.10 (0.66)
Social Support (Peers)	9.44 (3.39)	8.92 (3.60)	0.352	-0.52 (2.66)	8.10 (2.92)	8.37 (2.53)	0.026	0.27 (2.12)
Social Support (Family)	15.42 (4.03)	15.17 (5.50)	0.082	-0.25 (3.08)	15.40 (4.90)	14.70 (4.13)	0.843	-0.70 (3.33)
Self-Efficacy	30.20 (6.73)	28.56 (7.68)	0.737	-1.64 (5.57)	28.62 (6.20)	27.52 (8.17)	0.871	-1.10 (4.04)
Perceived Physical Competence	22.08 (4.56)	22.60 (4.64)	0.532	0.52 (2.62)	19.28 (5.44)	19.28 (5.48)	0.372	0.00 (1.83)
**Health Variables**								
Height (m)	1.65 (0.07)	1.66 (0.07)	0.007	0.00 (0.02)	1.63 (0.06)	1.65 (0.07)	<0.001	0.01 (0.05)
Weight (kg)	51.46 (5.65)	51.58 (5.79)	0.002	0.13 (1.78)	52.57 (6.45)	51.93 (6.44)	<0.001	-0.64 (1.91)
Body Mass Index (kg/m ^2^)	21.78 (5.62)	21.75 (5.64)	0.031	-0.03 (0.98)	22.47 (6.55)	21.86 (6.80)	<0.001	-0.62 (1.12) *
General Fitness	4.20 (0.65)	4.20 (0.87)	0.002	0.00 (0.76)	3.82 (1.12)	3.82 (1.12)	<0.001	0.00 (0.47)
Cardiorespiratory Fitness	3.80 (0.82)	3.92 (0.86)	0.040	0.12 (0.97)	3.43 (1.20)	3.75 (1.24)	<0.001	0.32 (0.66) *
Muscular Strength	3.72 (0.84)	3.84 (0.85)	<0.001	0.12 (0.60)	3.29 (0.98)	3.18 (1.02)	<0.001	-0.11 (0.63)
Speed/Agility	4.28 (0.79)	4.12 (0.78)	0.003	-0.16 (0.75)	3.34 (1.14)	3.52 (0.95)	<0.001	0.17 (0.71)
Flexibility	3.32 (0.99)	3.40 (0.96)	0.011	0.08 (1.08)	3.03 (1.02)	2.90 (1.05)	<0.001	-0.14 (1.03)
Somatic Health Complaints	.78 (0.95)	.82 (0.89)	<0.001	0.04 (0.37)	1.01 (1.00)	0.82 (0.94)	0.005	-0.19 (0.57)
Psychological Health Complaints	1.20 (1.03)	1.17 (1.17)	0.969	-0.03 (0.54)	1.40 (0.98)	1.15 (0.93)	0.028	-0.25 (0.52) *
Overall Health Complaints	7.92 (7.17)	7.96 (7.32)	0.168	0.04 (2.51)	9.62 (7.37)	7.83 (7.07)	0.001	-1.79 (3.65) *
**Wellbeing Variables**								
Wellbeing	51.24 (8.38)	51.52 (8.01)	0.693	0.28 (6.15)	44.79 (8.62)	45.62 (9.10)	0.340	0.83 (5.14)
Life Satisfaction	79.62 (18.76)	77.83 (22.77)	<0.001	-1.79 (6.74)	75.14 (19.60)	74.68 (20.94)	0.368	-0.46 (6.30)
**Covariates**								
Family Affluence	10.28 (2.30)	10.28 (2.19)	0.270	0.00 (1.35)	9.57 (2.37)	9.50 (2.48)	0.111	-0.07 (0.69)
Physical Impairment	3.96 (0.20)	3.88 (0.33)	<0.001	-0.08 (0.28)	3.86 (0.35)	3.86 (0.35)	<0.001	0.00 (0.18)

*Significant differences ( *p* <.05) were found between trial 1 and trial 2, paired-samples *t-*test. Abbreviations: Physical Activity Questionnaire for Adolescents (PAQ-A), Moderate to Vigorous Physical Activity (MVPA), Standard Deviation (SD), Time Point 1 (T1), Time Point 2 (T2), Meter (m), Kilograms (kg).

**Table 3.** Relative and absolute reliability indices for the physical activity behavior, health and wellbeing variables in male and female groups.

	Males					Females				
	ICC (95% CI)	CV	P	LOA (±1.96 SD)	Cronbach Alpha Coefficient	ICC (95% CI)	CV	P	LOA (±1.96 SD)	Cronbach Alpha Coefficient
**Physical Activity** ** Behavior Variables**										
PAQ-A	0.849 (.647-.935)	7.68%	0.987	1.016 to -0.983	0.843	0.947 (.889-.975)	7.33%	0.698	0.710 to -0.660	0.946
MVPA	0.830 (.612-.925)	18.69%	0.831	2.778 to -2.658	0.825	0.944 (.882-.973)	13.55%	0.167	1.714 to -1.314	0.945
Sedentary Behavior Weekday	0.779 (.449-.911)	8.74%	0.823	0.950 to -0.903	0.771	0.898 (.774-.953)	6.31%	0.984	0.577 to -0.575	0.894
Sedentary Behavior Weekend	0.777 (.474-.906)	9.90%	0.563	1.086 to -0.958	0.772	0.863 (.705-.936)	7.61%	0.526	0.762 to -0.863	0.860
Overall Sedentary Behavior	0.798 (.499-.918)	8.49%	0.610	0.969 to -0.863	0.792	0.938 (.862-.972)	5.48%	0.421	0.449 to -0.529	0.937
Intention to be Physically Active	0.557 (.045-.800)	16.33%	0.076	1.717 to -2.517	0.579	0.848 (.682-.927)	11.13%	0.326	1.565 to -1.298	0.848
Enjoyment of School	0.808 (.571-.915)	11.45%	0.183	2.282 to -1.722	0.813	0.861 (.702-.935)	10.91%	1.000	1.656 to -1.656	0.857
Enjoyment of Physical Education	0.855 (.673-.936)	7.97%	0.538	1.175 to -1.335	0.852	0.902 (.792-.954)	6.70%	0.745	1.144 to -1.075	0.900
Social Support (Friends)	0.824 (.588-.924)	5.07%	1.000	1.156 to -1.156	0.817	0.810 (.603-.909)	6.54%	0.415	1.197 to -1.397	0.808
Social Support (Peers)	0.831 (.621-.925)	18.29%	0.339	4.700 to -5.740	0.831	0.825(.634-.917)	13.76%	0.496	4.414 to -3.881	0.823
Social Support (Family)	0.890 (.745-.952)	13.16%	0.695	5.791 to -6.291	0.886	0.842 (.671-.924)	12.71%	0.260	5.834 to -7.234	0.843
Self-Efficacy	0.819 (.595-.919)	12.68%	0.154	9.271 to -12.551	0.825	0.913 (.815-.959)	10.01%	0.152	6.812 to -9.019	0.916
Perceived Physical Competence	0.912 (.803-.961)	6.06%	0.330	5.647 to -4.607	0.912	0.972 (.940-.987)	5.92%	1.000	3.591 to -3.591	0.971
**Health Variables**										
Height	0.976 (.944-.989)	0.57%	0.485	0.048 to -0.042	0.975	0.863 (.715-.934)	0.86%	0.215	0.107 to -0.085	0.890
Weight	0.976 (.945-.990)	1.53%	0.734	3.608 to -3.358	0.975	0.976 (.947-.989)	1.58%	0.086	3.099 to -4.385	0.978
BMI	0.993 (.983-.977)	2.10%	0.861	1.891 to -1.960	0.992	0.991 (.974-.996)	2.49%	0.007 *	1.575 to -2.808	0.993
General Fitness	0.676 (.251-.858)	7.43%	1.000	1.497 to -1.497	0.667	0.956 (.904-.979)	3.69%	1.000	0.924 to -0.924	0.954
Cardiorespiratory Fitness	0.504 (-.140-.782)	11.54%	0.543	2.024 to -1.784	0.497	0.904 (.766-.958)	12.74%	0.017 *	1.634 to -0.991	0.918
Muscular Strength	0.856 (.678-.936)	6.73%	0.327	1.296 to -1.056	0.856	0.891 (.765-.949)	8.12%	0.375	1.125 to -1.340	0.890
Speed/Agility	0.708 (.347-.871)	8.78%	0.294	1.302 to -1.622	0.710	0.868 (.722-.938)	11.62%	0.202	1.565 to -1.220	0.871
Flexibility	0.567 (.000-.811)	15.31%	0.714	2.191 to -2.031	0.558	0.676 (.309-.848)	15.62%	0.475	1.872 to -2.148	0.672
Somatic Health Complaints	0.959 (.906-.982)	52.29%	0.597	0.771 to -0.691	0.957	0.859 (.784-.953)	42.46%	0.084	0.927 to -1.306	0.906
Psychological Health Complaints	0.938 (.859-.973)	54.67%	0.784	1.032 to -1.092	0.935	0.904 (.767-.958)	27.93%	0.017 *	0.776 to -1.280	0.919
Overall Health Complaints	0.970 (.933-.987)	41.56%	0.937	4.956 to -4.876	0.969	0.919 (.801-.964)	32.28%	0.013 *	5.358 to -8.945	0.932
**Wellbeing Variables**										
Wellbeing	0.842 (.638-.930)	6.34%	0.822	12.329 to -11.769	0.836	0.909 (.807-.957)	6.06%	0.393	10.905 to -9.249	0.908
Life Satisfaction	0.971 (.933-.987)	4.15%	0.206	11.426 to -15.009	0.972	0.976 (.948-.989)	4.97%	0.700	0.975	11.880 to -12.808
**Covariates**										
Family Affluence	0.904 (.780-.958)	5.07%	1.000	4.613 to -4.614	0.900	0.980 (.958-.990)	4.14%	0.601	1.289 to -1.422	0.979
Physical Impairment	0.647 (.222-.842)	1.62%	0.161	0.463 to -0.623	0.657	1.000 (0.00-0.00)	0.00%	1.000	0.358 to -0.358	1.000

*Significant differences ( *p* <.05) were found between trial 1 and trial 2, paired-samples *t*-test. Intraclass Correlation Coefficient: <.50 (poor agreement); 0.50-0.75 (moderate agreement); 0.75-0.90 (good agreement); >0.90 (excellent agreement) Abbreviations: Physical Activity Questionnaire for Adolescents (PAQ-A), Moderate to Vigorous Physical Activity (MVPA), Standard Deviation (SD), Intraclass Correlation Coefficient (ICC), Confidence Interval (CI), Limits of Agreement (LOA), Coefficient of Variation (CV).

**Figure 1.**
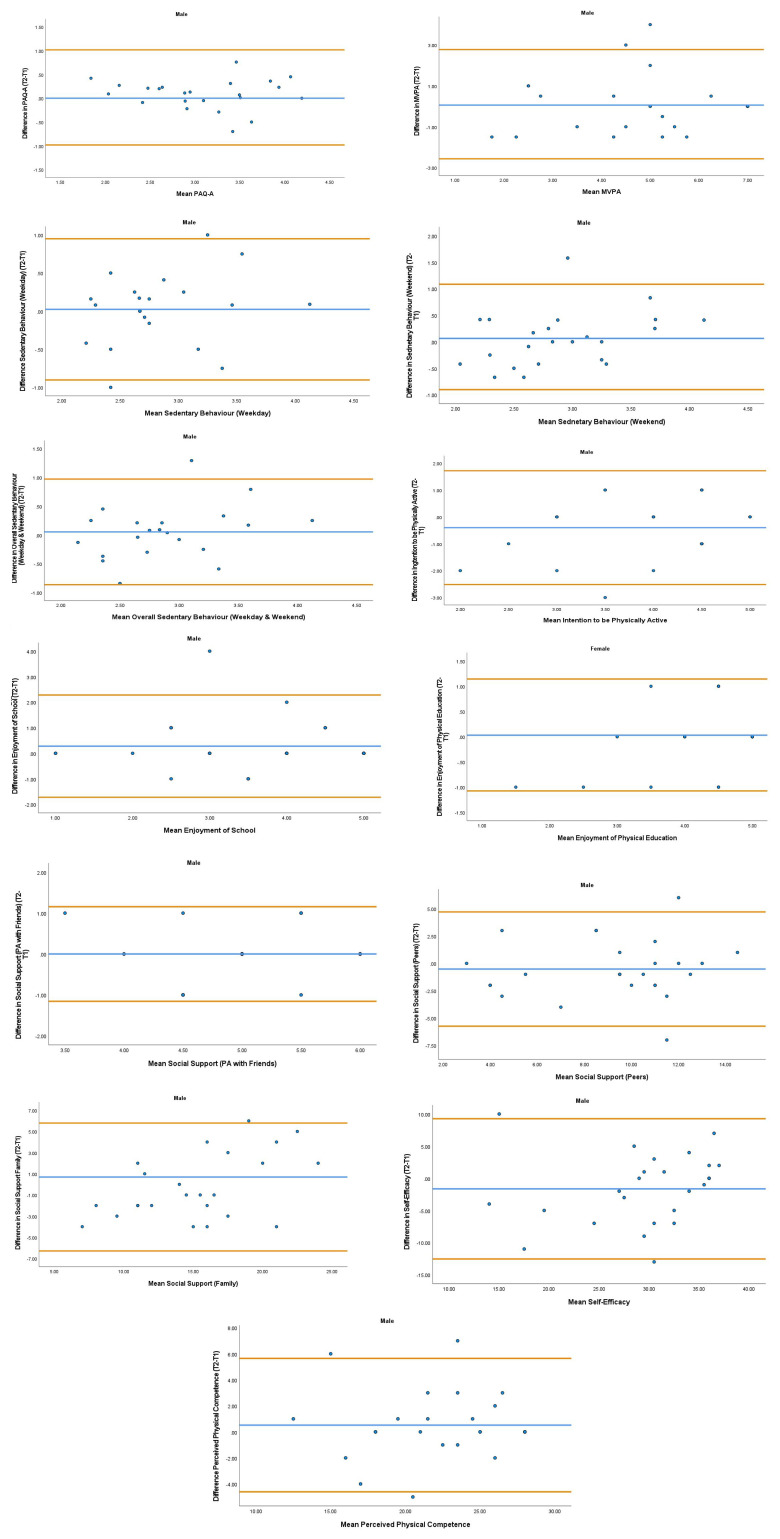
Bland-Altman Polts for Male Physical Activity Behavior Variables. Notes: The central blue line represents the mean differences between the T2 and the T1; the upper and lower orange lines represents the upper and lower 95% limits of agreement (means differences ± 1.96 SD of the differences). Variable protocols and the PABHAW questionnaire are available in the methods section and extended data of this manuscript. Abbreviations: Physical Activity (PA), Physical Activity Questionnaire for Adolescents (PAQ-A), Moderate to Vigorous Physical Activity (MVPA), Time Point 1 (T1), Time point 2 (T2).

The overall mean coefficient of variation was marginally lower for females (10.19%) than males (13.01%). Females had a lower coefficient of variation in 19/28 variables. For the physical activity behavior variables, the mean coefficient of variation was lower in females (9.07%) in comparison to males (11.12%). Similarly, the health variables reported a mean coefficient of variation that was lower in females (14.49%) in comparison to males (18.41%). The mean coefficient of variation associated with the wellbeing variables was consistent for both females (5.52%) and males (5.25%). Regarding the covariates, the mean coefficient of variation also reported lower results in females (0.80%) in comparison to males (3.35%). In addition, the combined mean Cronbach alpha coefficients were higher for girls (0.908) than boys (0.821). The blue central line in each Bland-Altman Plots ( Figure 1– Figure 6) demonstrated systematic error and was reported close to zero for each of the outcome variables. Visual inspection of the Bland-Altman plots indicated the data to be homoscedastic. That is, the presence of heteroscedasticity was not illuminated.

**Figure 2.**
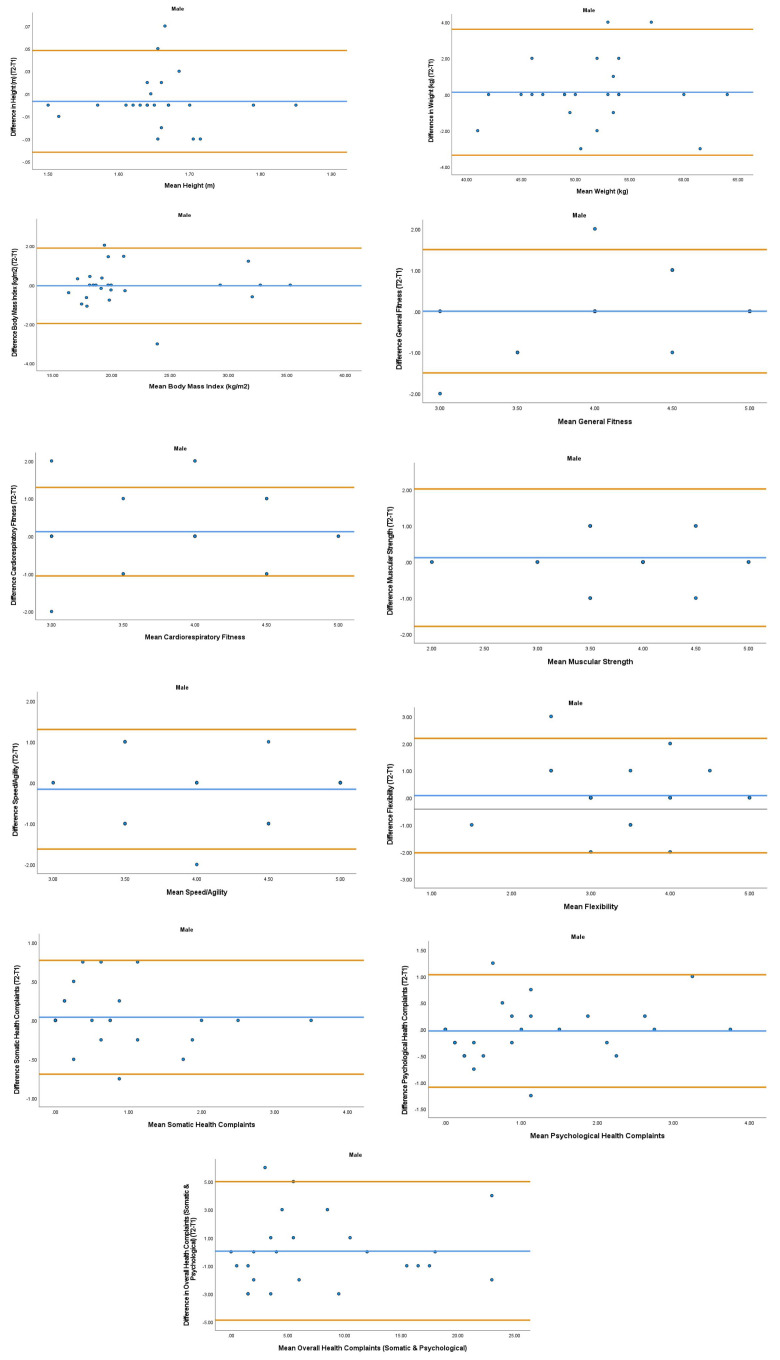
Bland-Aitman Plots for Male Health Variables. Notes: The central blue line represents the mean differences between the T2 and the T1; the upper and lower orange lines represent the upper and lower 95% limits of agreement (means differences ± 1.96 SD of the differences). Variable protocols and the PABHAW questionnaire are available in the methods section and extended data of this manuscript. Abbreviations: Physical Activity (PA), Moderate to Vigorous Physical Activity (MVPA), Time Point 1 (T1), Time Point 2 (T2).

**Figure 3.**
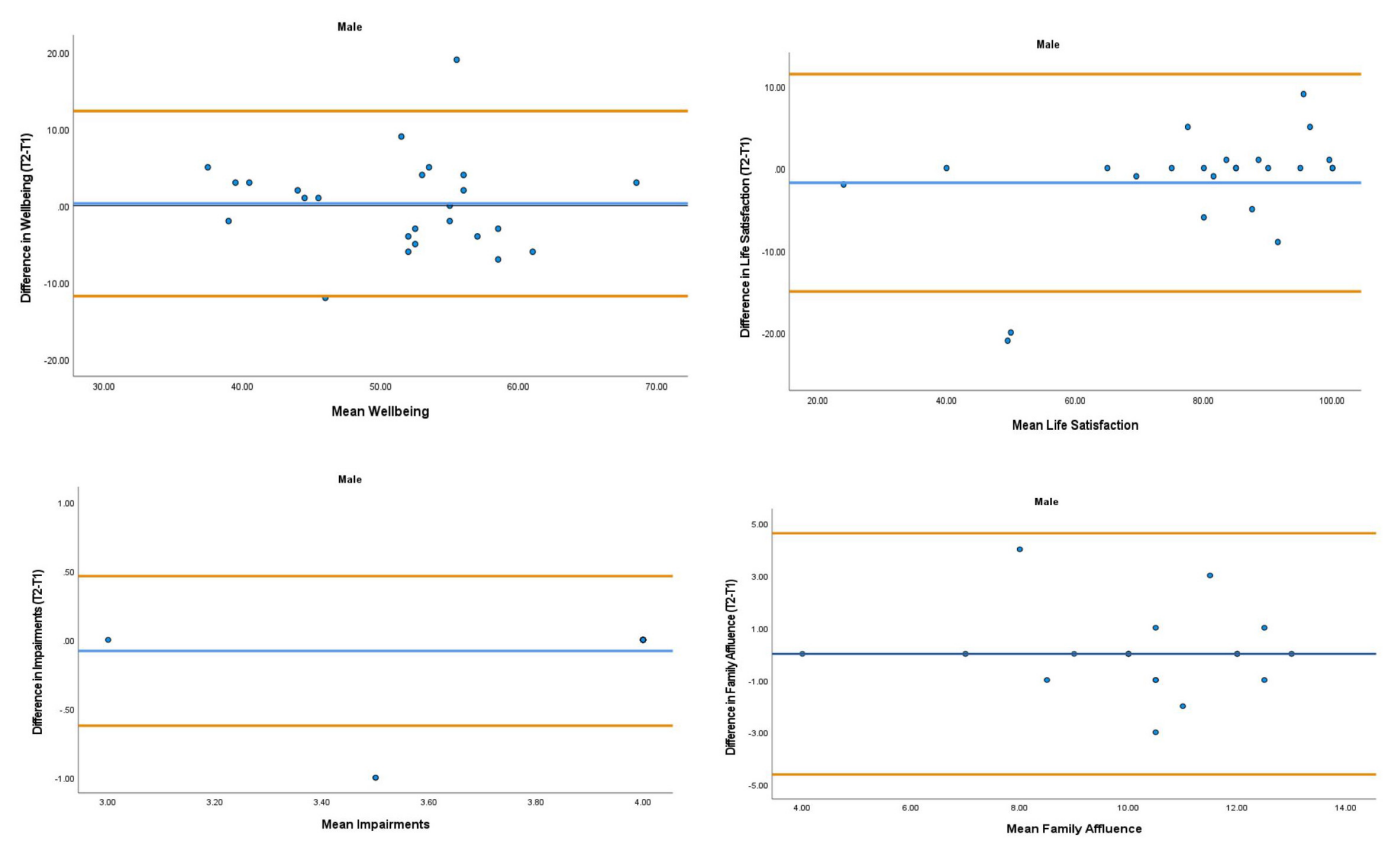
Bland-Altman Plots for Male Wellbeing Variables and Covariates. Notes: The central blue line represents the mean differences between the T2 and the T1; the upper and lower orange lines represent the upper and lower 95% limits of agreement (means differences ± 1.96 SD of the differences). Variable protocols and the PABHAW questionnaire are available in the methods section and extended data of this manuscript. Abbreviations: Physical Activity (PA), Moderate to Vigorous Physical Activity (MVPA), Time Point 1 (T1), Time Point 2 (T2).

**Figure 4.**
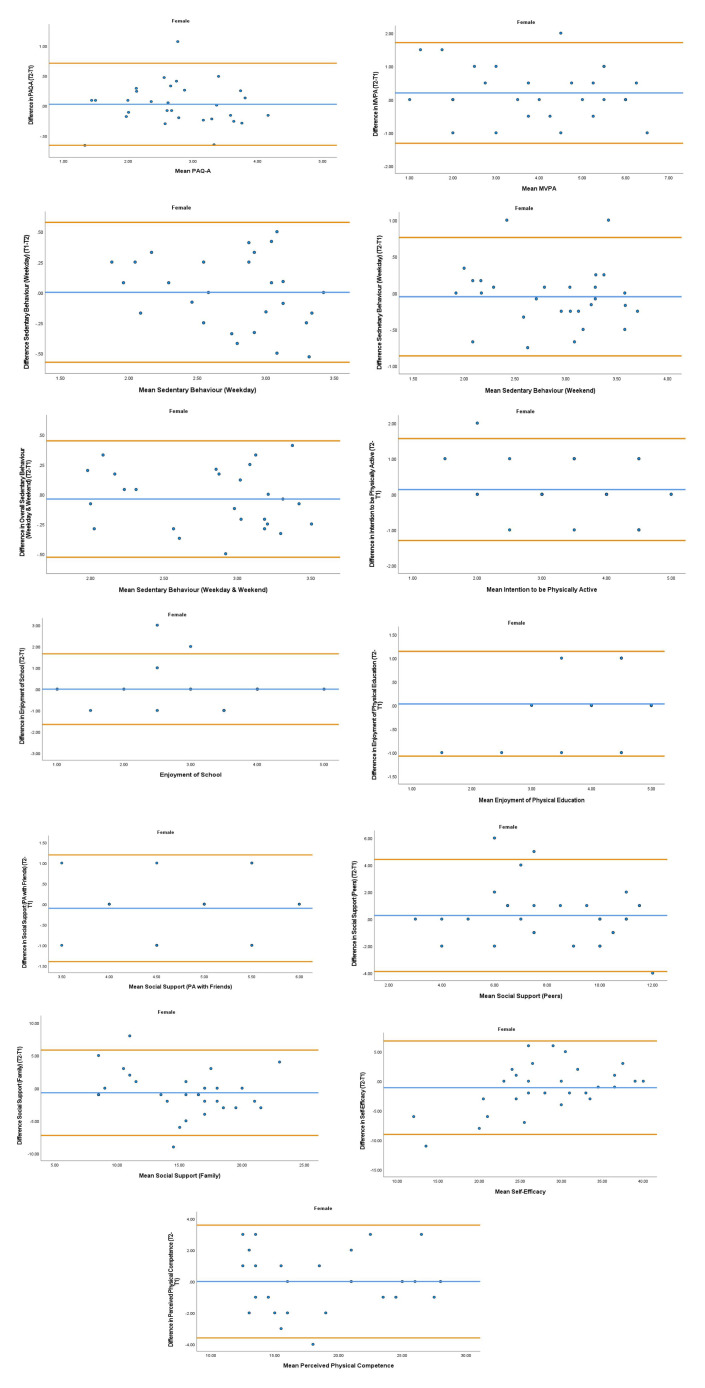
Bland-Altman Plots for Female Physical Activity Behavior Variables. Notes: The central blue line represents the mean differences between the T2 and the T1; the upper and lower orange represent the upper and lower 95% limits of agreement (means differences ± 1.96 SD of the differences). Variable protocols and the PABHAW questionnaire are available in the methods section and extended data of this manuscript. Abbreviations: Physical Activity (PA), Physical Activity Questionnaire for Adolescents (PAQ-A), Moderate to Vigorous Physical Activity (MVPA), Time Point 1 (T1), Time Point 2 (T2).

**Figure 5.**
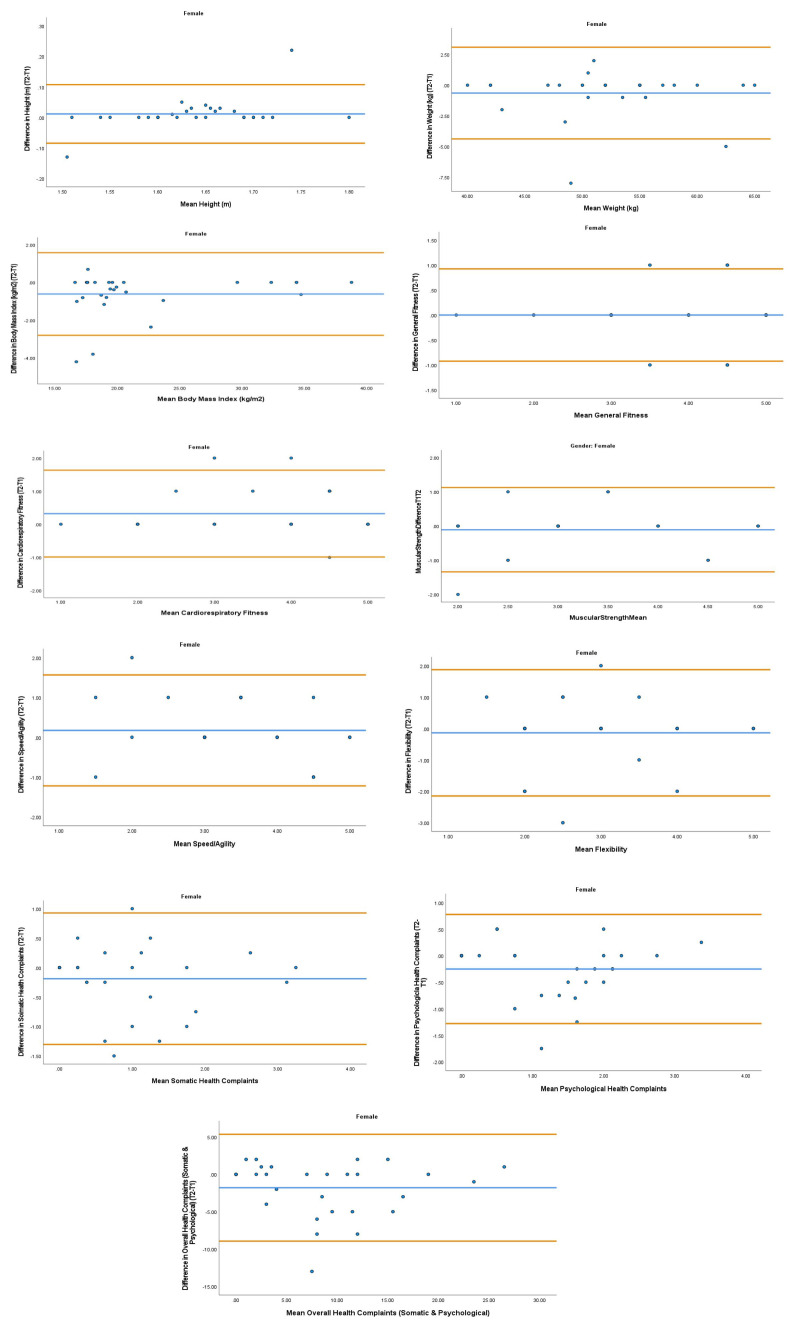
Bland-Altman Plots for Female Health Variables. Notes: The central blue line represents the mean differences between the T2 and the T1; the upper and lower orange lines represent the upper and lower 95% limits of agreement (means differences ± 1.96 SD of the differences). Variable protocols and the PABHAW questionnaire are available in the methods section and extended data of this manuscript. Abbreviations: Physical Activity (PA), Moderate to Vigorous Physical Activity (MVPA), Time Point 1 (T1), Time Point 2 (T2).

**Figure 6.**
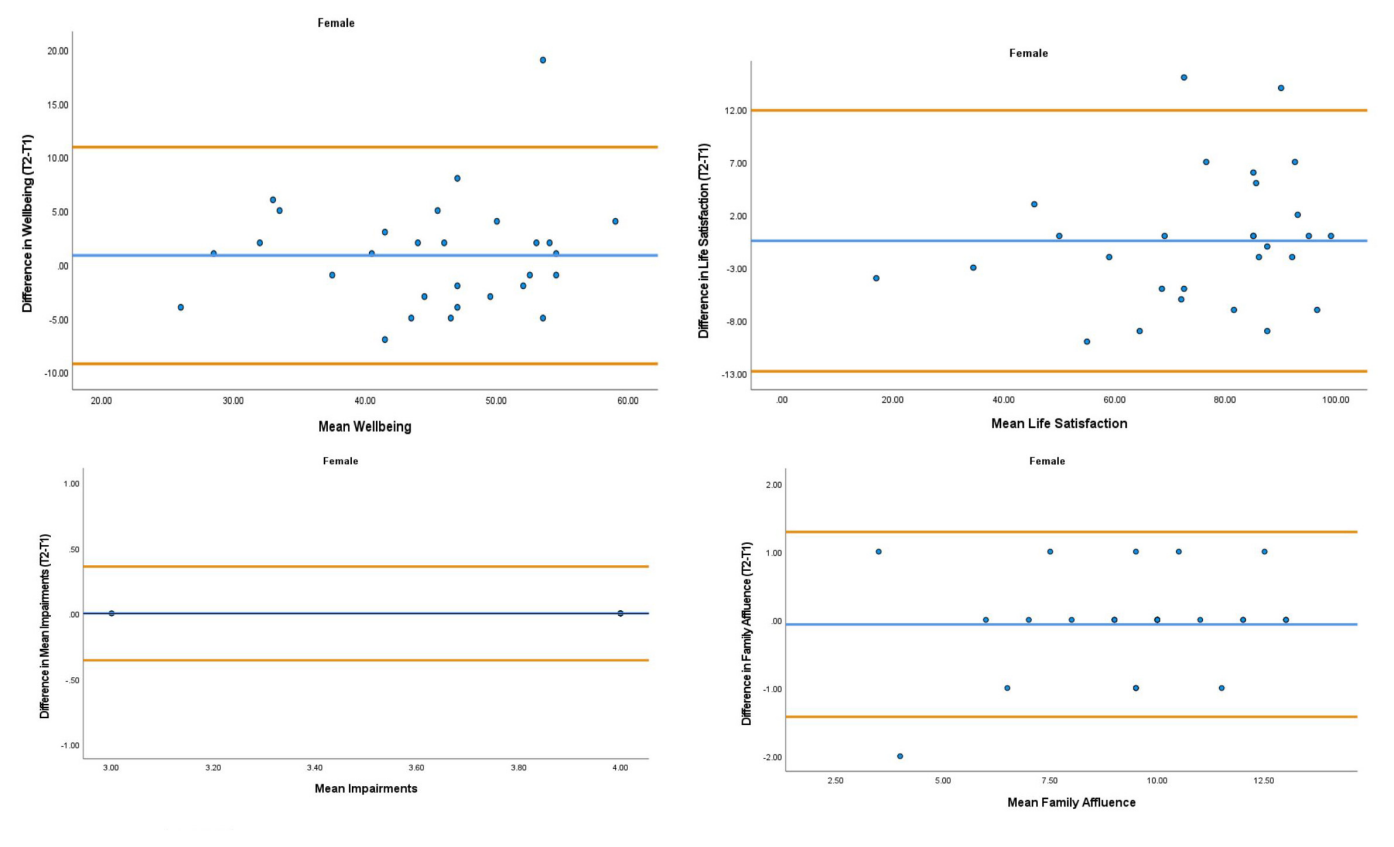
Bland-Altman Plots for Female Wellbeing Variables and Covariates. Notes: The central blue line represents the mean differences between the T2 and the T1; the upper and lower orange lines represent the upper and lower 95% limits of agreement (means differences ± 1.96 SD of the differences). Variable protocols and the PABHAW questionnaire are available in the methods section and extended data of this manuscript. Abbreviations: Physical Activity (PA), Moderate to Vigorous Physical Activity (MVPA), Time Point 1 (T1), Time Point 2 (T2).

## Discussion

The aim of this study was to examine the test-retest reliability of the PABHAW questionnaire, administered by teachers in school settings and utilized to estimate physical activity behavior, health and wellbeing in adolescent populations. The analysis in the current study offers pertinent insights in to the quality of the questionnaire when administered in school settings where adolescents spend a high proportion of their waking day ( Hobin *et al.*, 2017). Reliability was explored using relative (correlation coefficients) and absolute (intertest differences, coefficient of variation, Bland-Altman plots, limits of agreement) indices. Previous studies have established validity and reliability in some of the variables contained in the PABHAW questionnaire, however, this is the first study to combine a multitude of these variables in to a single questionnaire and assess the reliability via test-retest analysis. The combined responses of the adolescents were utilized to demonstrate the reliability of the PABHAW questionnaire and provide a foundation for its use in a national study that will examine the impact of different levels of typical school provision of physical education, physical activity and sports on adolescent physical activity behaviors, health and wellbeing in the Republic of Ireland. The findings gleaned from the current study illustrate the PABHAW questionnaire to be reliable. However, further research that confirms the reliability of BMI, cardiorespiratory fitness, psychological and overall health complaints in females may be warranted. Key findings from the current study will be contextualized within the framework of prior research outcomes and disparities will be discussed.

### Physical activity behavior

Reliability indices for moderate to vigorous physical activity (MVPA) were excellent for females (0.944) and good for males (ICC = 0.830). This is consistent with Prochaska *et al.* (2001) who found good test-retest reliability (ICC = 0.88) in a sample of 42 adolescents aged 13.9 ( *±* 1.7) years. Notably, these findings pertain to same day test-retest relative reliability indices, with further findings illuminating a decline of intraclass correlation values for retest up to one month (ICC = 0.53). A review of 17 studies that investigated the reliability of self-report physical activity instruments in adolescent populations corroborated these findings with stronger reliability found on same day retests ( Sallis & Saelens, 2000). Similar findings for retests up to one month, in adolescent populations, indicated moderate relative reliability indices for the same MVPA activity items in Vietnam (ICC = 0.58), Slovakia (ICC = 0.51) and the Czech Republic (ICC = 0.53) ( Bobakova *et al.*, 2015; Kohoutek *et al.*, 2022). Comparatively, a test-retest study in Poland that utilized the same retest period as the current study (1 week), illuminated excellent intraclass correlations (ICC = 0.98) ( Bobakova *et al.*, 2015). It is considered that shorter retest periods “could cause the carryover effects due to memory, practice or mood…whereas a longer interval increases the risk of a change in the condition” ( Bobakova *et al.*, 2015 p63). Therefore, the evidence indicates that a shorter retest period of one week or less may be the ideal timeframe to optimize reliability of the MVPA items and may prove pertinent in the wider context of MVPA measurement, in line with the World Health Organization physical activity recommendations ( 2022b).

The current study is the first to report the reliability of the PAQ-A ( Kowalski *et al.*, 2004) among Irish adolescents, in school settings ( Kowalski *et al.*, 2004). Acceptable relative and absolute reliability indices for the PAQ-A were reported to be for both males (ICC = 0.849; CV = 7.68%) and females (ICC = 0.947; CV = 7.33%). A near-universal agreement regarding the reliability of the PAQ-A is highlighted in the literature. A two-week test-retest study by Aggio *et al.* (2016) conducted on 160 English adolescents aged 14.5 ( *±* 1.7) years, found the PAQ-A to demonstrate acceptable reliability (ICC = 0.78). Notably, item 1 of the PAQ-A was modified to reflect activities and sports that were representative of the sample, similar to the current study. This is consistent with another test-retest reliability study that demonstrated acceptable reliability (ICC = 0.78) among 110 Thai adolescents over two weeks, in which the PAQ-A was also modified to reflect the activities closely aligned with Thai culture ( Pratanaphon *et al.*, 2020). In addition, test-retest reliability of the PAQ-A, assessed in Poland ( Wyszynska *et al.*, 2019), Turkey ( Aygun Polat *et al.*, 2021), Malaysia ( Koh *et al.*, 2020), Spain ( Martinez-Gomez *et al.*, 2009) and India ( Rahayu *et al.*, 2022) with rest intervals of 1–2 weeks, further corroborate the findings in the current study. However, a five-week test-retest study by Andarge *et al.* (2021) demonstrated poor relative reliability indices (ICC = 0.34) among 110 adolescents of 14–19. This inconsistency is attributed to the prolonged test re-test interval in comparison to the timeframe illuminated in the aforementioned studies. Therefore, test-retest intervals of two weeks or less when assessing the reliability of the PAQ-A, in accordance with the current evidence, may be optimal. Notably, there is a dearth of coefficient of variation values reported in test-retest reliability studies in the literature which should also be considered to contrast absolute and relative reliability indices.

Relative and absolute reliability patterns for the intention to be physically active scale ( Godin & Shepard, 1986); ranged from moderate in males (ICC = 0.557; CV = 16.33%) to good in females (ICC = 0.848; CV = 11.13%). Overall, relative indices were considered among the lowest of the physical activity behavior variables. There is a paucity of research examining the validity and reliability of the intention to be physically active scale, as confirmed by Brown *et al.* (2009) in a systematic literature review on instruments to assess potential mediators of physical activity in youth. A test-retest study conducted by Saunders *et al.* (1997) on 558 5 ^th^ grade students (10–11 years), found the intention to be physically active scale demonstrated acceptable reliability (r = 0.63). However, there was a retest period of one year and alternative statistical analysis were utilized (Pearson product-moment correlation coefficient). In addition, the data was collected more than 25 years previous. Identical findings using Pearsons’s product-moment correlation coefficients (r = 0.63) were found over a one-week timeframe by Pate *et al.* (2003). This is inconsistent with Godin and Shephard (1986) who found greater relative relatability indices for the intention to be physically active scale with a two-week retest protocol (r = 0.87). However, these data were collected in excess of 35 years ago, further illuminating the need for the current study. Findings in the current study are however in corroboration with Marcoux *et al.* (1999) who found comparable relative reliability patterns (ICC = 0.60) for the intention to be physically active scale. However, the timeframe consisted of a 6-month retest protocol, and it is unclear if the instrument is precisely the intention to be physically active scale or a modified version. Notably, there is a dearth of evidence that pertains to reliability patterns split for males and females which may also be considered in future research to contrast the current findings. Furthermore, the aforementioned studies solely presented relative correlation coefficient indices, despite the acknowledged limitations of this approach, as previously outlined.

The correlation between the social influence’s scales ( Reynolds *et al.*, 1990), perceived physical competence and self-efficacy, with the intention to be physically active in adolescents, is a frequent finding in the literature and are some of the most important predictors of physical activity ( Erdvik *et al.*, 2014; Fernandez-Rio *et al.*, 2018; Hamilton *et al.*, 2017; Saunders *et al.*, 1997; Trigueros *et al.*, 2019; Xiao *et al.*, 2019). Relative reliability indices for peer and family support in the current study were good for both males (ICC = 0.831; 0.890) and females (ICC = 0.825; 0.842). This is consistent with Prochaska *et al.* (2002) who also found good test-retest reliability for peer (ICC = 0.88) and family (ICC = 0.86) support over a two-week period. A test-retest study examining the reliability of perceived physical competence with a four-week interval reported excellent reliability (ICC = .0.90) ( Muris *et al.*, 2003). These findings are corroborated by the current study that found excellent relative reliability indices for perceived physical competence, as defined by Harter (1982), in both males (ICC = 0.912) and females (ICC = 0.972). Notably, absolute indices for perceived physical competence were among some of the lowest of the physical activity behavior variables for males (CV = 6.06%) and females (CV = 5.92%). However, the compatibility of test-retest protocols to measure perceived physical competence is contested as “the investigator may be sensitive to the potential for actual changes over time, which renders time 1 versus time 2 comparisons problematic, as an index of reliability to assess psychometric adequacy” ( Harter, 2012 p12). Despite marginally higher coefficient of variation values for the self-efficacy scale ( Saunders *et al.*, 1997) in males (CV = 12.68%) and females (CV = 10.01%), intraclass correlation coefficients indicated good (ICC = 0.819) and excellent (ICC = .913) indices in males and females respectively. These relative indices are consistent with Motl *et al.* (2000) who found acceptable reliability patterns (0.66) for the self-efficacy scale over a period of one year, albeit these findings are of moderate strength comparatively. The aforementioned variance may be due to alternative relative coefficient statistical analysis utilized in this study or differing protocols pertaining to the timeframe between T1 and T2. Once more, a lack of absolute indices regarding test-retest reliability patterns associated with self-efficacy are clear. 

Overall, the physical activity behavior variables are considered reliable.

### Health

The combined mean coefficient of variation for the health variables was lower in females (14.49%) in comparison to males (18.41%). However, it should be noted that the acute nature of the items included in the Health Behavior in School-Aged Children’s subjective health complaints symptoms checklist (e.g., headache, stomach ache, feeling nervous, feeling dizzy) may account for the considerably higher combined coefficients of variation values in comparison to physical activity behavior and wellbeing. Notably, a significant intertest difference was reported for cardiorespiratory fitness ( *p* = 0.017) psychological ( *p* = 0.017) and overall health complaints ( *p* = 0.013) suggesting a decline in reliability in females. Despite the high coefficient of variation in males and females respectively for somatic health complaints (52.29%; 42.46%), psychological health complaints (54.67%; 27.93%) and overall health complaints (41.56%; 32.28%), relative correlation coefficient indices were excellent in males (ICC = 0.959; ICC = 0.938; ICC = 0.970) and good-excellent in females (ICC = 0.859; ICC=.904; ICC = 0.919). The variance in the scores observed for both males and females may underpin this inconsistency and further emphasizes the need to utilize both relative and absolute reliability indices when examining reliability as described by Atkinson & Nevill (1998). Interestingly, relative reliability analysis by Kohoutek *et al.* (2022) found subjective health complaints to be the lowest of a range of measures in Vietnamese adolescents (ICC = >0.43) while higher levels of reliability (ICC = >.061) were illuminated in Norwegian adolescents ( Haugland & Wold, 2001). This suggests the potential impact of factors such as cultural differences in the consistency of respondents ( Lansford *et al.*, 2010; Weiss *et al.*, 2014). It is noteworthy that the aforementioned studies performed test-retest reliability analysis at the item level as opposed to two subscales (somatic health complaints and psychological health complaints) in accordance with Potrebny *et al.* (2019) as previously outlined in the methods section of this study.

Reliability patterns ranged between moderate-excellent for males and females on the five items included in the International Fitness Scale ( Ortega *et al.*, 2011); general fitness (ICC = 0.676; ICC = 0.956), cardiorespiratory fitness (ICC = 0.504; ICC = 0.904) muscular strength (ICC = 0.856; ICC = 0.891), speed (ICC = 0.708; ICC = 0.868), flexibility (ICC = 0.567; ICC = 0.679). Interestingly, more consistent reliability patterns that ranged between moderate-good were found in the physical fitness domain (e.g., cardiovascular endurance, muscular strength/endurance, flexibility, speed, agility) of the Physical Literacy in Children Questionnaire for males (ICC = 0.89) and females (ICC = 0.69) ( Barnett *et al.*, 2022). Notably, a key methodological difference between the two studies pertain to the timeframe with one week between T1 and T2 in the current study, a common testing interval in the literature ( Polit, 2014; Ramirez-Velez *et al.*, 2017), and two weeks or more in the latter ( Barnett *et al.*, 2022). It is considered that a shorter testing interval “can mean the participant is responding with regard their memory”, which may lead to inflated reliability patterns ( Barnett *et al.*, 2022 p10). Therefore, future studies may consider a two-week testing interval as a viable strategy to optimize the validity of physical fitness test-retest data. Coefficient of variation values for the International Fitness Scale variables were similar for both males and females, respectively. It is considered that cost effectiveness, ease of administration and participant compliance due to the less invasive nature of self-report measures may be key advantages when measuring physical fitness in school settings ( Saw *et al.*, 2015).

Relative and absolute indices were excellent for anthropometric measures of height, weight and BMI in both males (ICC = 0.976; 0.976; 0.993; CV = 0.57%; 1.53%; 2.10%) and females (ICC = 0.863; 0.976; 0.991; CV = 0.86%; 1.58%; 2.49%), despite the observation of systemic bias in BMI in females ( *p* =.007). Notably, the participants had their height and weight measured by a parent/guardian at home, prior to completing the PABHAW questionnaire for T1 and T2, as previously outlined in the methods section of the current study. This suggests that objective measures of height and weight, administered by parents/guardians may present a cost effective, feasible alternative to measuring BMI in adolescent populations. However, O’Keeffe *et al.* (2019) utilized both student (ICC = .998; CV = 0.7%) and research-assistant (ICC = .999; CV = 0.6%) administered protocols to measure BMI and also found excellent relative and absolute reliability patterns. In addition, a reliability study on a range of health-related fitness indicators, including anthropometric measures in 80 adolescents, measured by physical education teachers, was found to be reliable ( Espana-Romero *et al.*, 2010). Moreover, measures of BMI administered by school nurses also indicated good reliability ( Stoddard *et al.*, 2008). Thus, research contrasting the measurement of BMI by a range of administrators, utilizing identical protocols is warranted. It is understood that “fitness testing can be a source of anxiety, fear and overwhelm for some students” ( Alfrey, 2024 p1; O’Keeffe *et al.*, 2021). Furthermore, weight evaluation in the presence of peers can lead to both negative experiences in school and physical activity participation ( Ladwig *et al.*, 2018; Sabiston *et al.*, 2014). Therefore, BMI evaluation at home, administered by a parent or guardian may be a suitable alternative. However, it must be noted that a substantial proportion of the literature illuminates the reliability of height and weight measures when conducted by trained administrators and the benefits of conducting these measures at least twice per time point to ensure accuracy, which was not the case in the current study ( Ahmed *et al.*, 1990; Allison *et al.*, 2020; O’Keeffe *et al.*, 2019; Vegelin *et al.*, 2003). Therefore, adequate familiarization protocols to appropriately train parents/guardians, should they lead BMI measurement, should be considered.

Overall, the health variables are considered reliable with the exception of BMI, cardiorespiratory fitness, psychological health complaints and overall health complaints in females.

### Wellbeing

Males had a minimally lower combined mean coefficient of variation value (5.24%) in comparison to females (5.51%) when examining the wellbeing variables (life satisfaction and wellbeing combined). In the context of wellbeing alone, estimated using the Warwick wellbeing scale ( Tennant *et al.*, 2007), relative intraclass correlation coefficient indices were also reported to be good for males (ICC = 0.842) and excellent for females (ICC = 0.909). This is consistent with Clarke *et al.* (2011), who reported acceptable relative indices (albeit a weaker association), via intraclass correlations (ICC = 0.66) when administered by teachers in school settings, among other studies ( Tennant *et al.*, 2007). Similar reliability patterns for life satisfaction, estimated via the Cantril ladder ( Cantril, 1965), demonstrated excellent relative reliability indices for both males (ICC = 0.971) and females (ICC = 0.976) and were further corroborated when comparing against absolute reliability indices (CV = 4.15%; CV = 4.97%). These findings are supported by a test- retest reliability study of 525 adolescent males and females that also found high relative reliability indices that were marginally lower in females (ICC = 0.79) than males (ICC = 0.80) ( Levin & Currie, 2014). Moreover, Pearson correlations by Muldoon *et al.* (2010) demonstrated the Cantril ladder to exhibit good reliability (r = 0.70) in school-aged children illuminating both the linear association and consistency of the measure across two time points, further emphasizing the validity of its use during this phase of life.

Overall, the wellbeing variables are considered reliable.

### Strengths and limitations

The current study is the first of its kind to conduct an examination into the test-retest reliability of a physical activity behavior, health and wellbeing questionnaire in adolescent populations, administered by teachers in school settings. The physical activity behavior, health and wellbeing questionnaire was assembled using variables with established validity and reliability and was administered using standardized guidelines. A detailed description of the protocols for each physical activity behavior, health and wellbeing item is included in the methods section of the current study. A mixed sample of both males and females was split for the analysis. A broad range of relative and absolute reliability indices were utilized to conduct a comprehensive analysis of the data. Lastly, recommendations regarding both optimal intervals between T1 and T2 and most suitable administrators are provided in the context of future research.

However, some limitations need to be considered. Due to the small convenience sample from three secondary schools located in the southern region of the Republic of Ireland, with a small age range, generalizability of the findings may be limited. Therefore, future studies may consider a more diverse sample to ensure a more detailed assessment of the reliability of the physical activity behavior, health and wellbeing questionnaire. Examinations of physical activity behavior, health and wellbeing were carried out by means of self-reported data with the exception of weight, height and body mass index. In addition, convergent reliability that compares many of the self-reported physical activity behavior, health and wellbeing questionnaire variables with equivalent gold standard measures (e.g., self-report activity versus accelerometer measured physical activity) was not conducted and may be considered in future research. Numerous studies in the literature highlight the importance of conducting measures of height and weight at least twice per time point to ensure accuracy ( Ahmed *et al.*, 1990; Allison *et al.*, 2020; O’Keeffe *et al.*, 2019; Vegelin *et al.*, 2003), a practice not followed in the current study.

## Conclusion

Measures to combat physical inactivity, ill health and wellbeing in adolescent populations are a public health priority. Therefore, the aim of this study was to examine the test-retest reliability of the physical activity behavior, health and wellbeing questionnaire, administered by teachers in school settings and utilized to estimate levels of physical activity behavior, health and wellbeing in adolescents. Although some research exists to support the reliability of questionnaires to measure physical activity behavior, health and wellbeing, there is a scarcity of research examining the reliability of tools to estimate all components as part of one questionnaire, utilizing a range of relative and absolute reliability indices. The current study found the physical activity behavior, health and wellbeing questionnaire to be a reliable measure of physical activity behavior, health and wellbeing in adolescent populations. However, it is recommended that further research is conducted on the reliability of the body mass index, cardiorespiratory fitness, psychological and overall health complaints measures in females. Overall, it is considered that the physical activity behavior, health and wellbeing questionnaire offers an accessible, cost-effective procedure to estimate key indicators of physical activity, behavior, health and wellbeing in adolescent populations.

## Data Availability

Figshare: Physical Activity Behavior, Health and Wellbeing Study - Test-Retest Dataset, https://doi.org/10.34961/researchrepository-ul.23985903.v2 ( Rocliffe *et al.*, 2023d). The project contains the following underlying data: Test-Retest Reliability of a Physical Activity Behavior, Health and Wellbeing Questionnaire in Adolescents SPSS Dataset.sav Figshare: Physical Activity Behavior, Health and Wellbeing Study – Questionnaire, https://doi.org/10.34961/researchrepository-ul.23985345.v2 ( Rocliffe *et al.*, 2023e). Data are available under the terms of the Creative Commons Attribution 4.0 International license (CC-BY 4.0).
