# Supplementary material for: Test-Retest Reliability of a Physical Activity Behavior, Health and Wellbeing Questionnaire in Adolescents
Source: Open Res Eur. 2024 Sep 5;3:154. Originally published 2023 Sep 19. [Version 3] doi: 10.12688/openreseurope.16535.3 (PMC11380079; doi:10.12688/openreseurope.16535.3)
Supplement: Peer review history

# Reviewer report: Reviewer response for version 3

**DOI:** 10.21956/openreseurope.19958.r43988

**Published:** 2024-09-06

**Authors:** Jittima Saengsuwan

**Affiliations:** 1 Department of rehabilitation medicine, Faculty of medicine, Khon Kaen University, Nai Mueang, Khon Kaen, Thailand

Thank you for addressing my queries.

If applicable, is the statistical analysis and its interpretation appropriate?

Yes

Is the study design appropriate and is the work technically sound?

Yes

Is the work clearly and accurately presented and does it engage with the current literature?

Yes

Are the conclusions drawn adequately supported by the results?

Yes

Are sufficient details of methods and analysis provided to allow replication by others?

Yes

Are all the source data and materials underlying the results available?

Yes

Reviewer Expertise:

neurological rehabilitation; exercise testing

I confirm that I have read this submission and believe that I have an appropriate level of expertise to confirm that it is of an acceptable scientific standard.

# Reviewer report: Reviewer response for version 3

**DOI:** 10.21956/openreseurope.19958.r37371

**Published:** 2024-09-06

**Authors:** Emmanuel Navarro-Flores, Daniel Lopez-Lopez

**Affiliations:** 1 Health Sciences, Universidade da Coruna, A Coruna, Galicia, Spain; 2 Department of Nursing, University of Valencia, Valencia, Spain

the authors have adressed all my requeriments in the correct way

If applicable, is the statistical analysis and its interpretation appropriate?

Yes

Is the study design appropriate and is the work technically sound?

Yes

Is the work clearly and accurately presented and does it engage with the current literature?

Partly

Are the conclusions drawn adequately supported by the results?

Yes

Are sufficient details of methods and analysis provided to allow replication by others?

Yes

Are all the source data and materials underlying the results available?

Yes

Reviewer Expertise:

Podiatry, Health related quality of life, Questionnaire

We confirm that we have read this submission and believe that we have an appropriate level of expertise to confirm that it is of an acceptable scientific standard.

# Reviewer report: Reviewer response for version 2

**DOI:** 10.21956/openreseurope.19425.r41781

**Published:** 2024-08-02

**Authors:** Jittima Saengsuwan

**Affiliations:** 1 Department of rehabilitation medicine, Faculty of medicine, Khon Kaen University, Nai Mueang, Khon Kaen, Thailand

- Thank you very much for your revisions. I would like to point out that some of the comments I made, which I regard as important, have not been adequately addressed. I would be grateful if you would kindly address these comments as follows:

 - You did not explain how the sample size was calculated. The term "convenient sampling" is not sufficient.

 - The figure still can be improved. I noticed discrepancies in subfigure size and unequal x and y-axis scales.

 - I suggest moving the p-value from the t-test and the Limits of Agreement (LoA) to Table 2 to enhance data clarity, as LoA is typically reported as MD ± 95% LoA. The authors showed the mean and standard deviation (SD) of Trials 1 and 2, along with the mean difference, all analyzed using a paired t-test in Table 2. However, the p-value is reported in Table 3, which separates related data that should ideally be presented together.

 - In addition, please specify whether the Shapiro-Wilk p-value was calculated from the first trial, the second trial, or the mean difference between trials.

If applicable, is the statistical analysis and its interpretation appropriate?

Yes

Is the study design appropriate and is the work technically sound?

Yes

Is the work clearly and accurately presented and does it engage with the current literature?

Yes

Are the conclusions drawn adequately supported by the results?

Yes

Are sufficient details of methods and analysis provided to allow replication by others?

Yes

Are all the source data and materials underlying the results available?

Yes

Reviewer Expertise:

neurological rehabilitation; exercise testing

I confirm that I have read this submission and believe that I have an appropriate level of expertise to confirm that it is of an acceptable scientific standard, however I have significant reservations, as outlined above.

# Reviewer report: Reviewer response for version 2

**DOI:** 10.21956/openreseurope.19425.r41780

**Published:** 2024-06-29

**Authors:** Emmanuel Navarro-Flores, Daniel Lopez-Lopez

**Affiliations:** 1 Department of Nursing, University of Valencia, Valencia, Spain; 2 Health Sciences, Universidade da Coruna, A Coruna, Galicia, Spain

Authors have addressed all my requirements in the correct way.

If applicable, is the statistical analysis and its interpretation appropriate?

Yes

Is the study design appropriate and is the work technically sound?

Yes

Is the work clearly and accurately presented and does it engage with the current literature?

Partly

Are the conclusions drawn adequately supported by the results?

Yes

Are sufficient details of methods and analysis provided to allow replication by others?

Yes

Are all the source data and materials underlying the results available?

Yes

Reviewer Expertise:

Podiatry, Health related quality of life, Questionnaire

We confirm that we have read this submission and believe that we have an appropriate level of expertise to confirm that it is of an acceptable scientific standard.

# Reviewer report: Reviewer response for version 1

**DOI:** 10.21956/openreseurope.17849.r38390

**Published:** 2024-04-18

**Authors:** Jittima Saengsuwan

**Affiliations:** 1 Department of rehabilitation medicine, Faculty of medicine, Khon Kaen University, Nai Mueang, Khon Kaen, Thailand

Overall, the manuscript is well written. I have some points as described below.

- Please recheck for the missing bracket, and add the unit (i.e., years) in the following sentence: A cross-sectional, mixed sample of 55 participants (45.5% males: Age, 13.94 (±.40) were included. 
- Please ensure that the unit (e.g., age) is added to Table 1 and throughout the manuscript.
- Regarding the expert stakeholder group, could the authors provide more information in the methods section? For example, how many participants are head physical education teachers, and how many are experts in the field (How was this defined? Years of practice?)?
- I noticed that there is missing data in your data file. The authors should specify 'n' for each variable that is not equal to the original number in the table. I suggest it might be easier for the authors to split the data into two tables, one for males and one for females. Each table should include all related outcomes (Test 1, Test 2, Mean Difference, p-value, LoA, CV, and ICC (95% CI)).
- In the Results section, please provide the full name of 'M' before using an abbreviation. If the authors intend to refer to 'mean difference', I would suggest using 'MD'. Additionally, please clarify the meaning of the number in parentheses; does it represent the standard deviation (SD)?
- I suggest moving the p-value from the t-test and Limits of Agreement (LoA) to Table 2 to enhance data clarity, as LoA is typically reported as MD ± 95% LoA.
- Height and weight could be omitted from the figure since the primary variable of interest is BMI.
- How was the sample size determined?
- Why do some figures have so few data points? For example, Fig 3 (Mean Impairments) and Fig 2 (Mean General Fitness).
- The figures could be improved. I noticed discrepancies in subfigure size and unequal x and y-axis scales. Additionally, each subfigure could be labeled as (a), (b), etc.
- There is no need to label Male/Female in every subfigure.

If applicable, is the statistical analysis and its interpretation appropriate?

Yes

Is the study design appropriate and is the work technically sound?

Yes

Is the work clearly and accurately presented and does it engage with the current literature?

Yes

Are the conclusions drawn adequately supported by the results?

Yes

Are sufficient details of methods and analysis provided to allow replication by others?

Yes

Are all the source data and materials underlying the results available?

Yes

Reviewer Expertise:

neurological rehabilitation; exercise testing

I confirm that I have read this submission and believe that I have an appropriate level of expertise to confirm that it is of an acceptable scientific standard, however I have significant reservations, as outlined above.

# Reviewer report: Reviewer response for version 1

**Published:** 2024-02-27

**Authors:** Emmanuel Navarro-Flores, Daniel Lopez-Lopez

**Affiliations:** 1 Department of Nursing, University of Valencia, Valencia, Spain; 2 Health Sciences, Universidade da Coruna, A Coruna, Galicia, Spain

I am grateful for the possibility to review this research study.

 This study supports novel information about the concurrent validity, test-retest reliability and usability of a Physical Activity Behavior,Â Health and Wellbeing Questionnaire in Adolescents.

 Â 

 This is an interesting aim with the quality of life scope.Â 

 This is a well-written manuscript with an important clinical message, and should be of great interest to the readers. However, from my point of view, the authors should include the following requirements

 Â 

 The redaction is clear and concise with appropriated scientific terms: physical Activity Behavior,Â Health and Wellbeing Questionnaire in Adolescents is a trend topic in the current research literature and may be a main focus of interest for readers

 Â 

 The sample size calculation, structured tables and methodology are adequate and provide important contents.

 Â 

 Therefore, this study may support considerations about the the concurrent validity, test-retest reliability and usability of Physical Activity Behavior,Â Health and Wellbeing Questionnaire in Adolescents.

 Â 

 On the other hand, the Introduction section may be improved by adding new information in order to provide an adequate state-of-the-art including some references. I suggest to include these references listed below to complete this requirementÂ 

 The Introduction may be improved adding new information in order to provide an adequate state-of-the-art. Furthermore, a hypothesis is lacking.

 Methods are well-designed with relevant and complete information.

 Discussion section may include future research studies secondary to the current findings of this study. Clinical considerations, limitations and overall discussion are well-presented, but future research may be useful in order to propose future research regarding this field.

 I suggest to include the following references to compare the achievement research with other similar prior researches (Navarro-Flores *et al.*, 2022 ^1^; Ahmadnezhad *et al.*, 2023 ^2^).Â I believe that it is necessary to include these references in order to improve the research evidence level quality. The suggested references are related to transcultural adaptation and validation of clinimetric tools

If applicable, is the statistical analysis and its interpretation appropriate?

Yes

Is the study design appropriate and is the work technically sound?

Yes

Is the work clearly and accurately presented and does it engage with the current literature?

Partly

Are the conclusions drawn adequately supported by the results?

Yes

Are sufficient details of methods and analysis provided to allow replication by others?

Yes

Are all the source data and materials underlying the results available?

Yes

Reviewer Expertise:

Podiatry, Health related quality of life, Questionnaire

We confirm that we have read this submission and believe that we have an appropriate level of expertise to confirm that it is of an acceptable scientific standard, however we have significant reservations, as outlined above.

## References

[1] : Repeatability and reliability of the diabetic foot self-care questionnaire in Arabic patients: A transcultural adaptation. *J Tissue Viability* .2022;31(1) : 10.1016/j.jtv.2021.06.007 62-68 10.1016/j.jtv.2021.06.007 34183224

[2] : *J Tissue Viability* .2023;32(2) : 10.1016/j.jtv.2023.02.009 255-261 10.1016/j.jtv.2023.02.009 36841714
